# Our Exchange Journals

**Published:** 1845-12-01

**Authors:** 


*Our Exchange Journals.*—One of the most agreeable circumstances connected with our editorial undertaking is the courtesy which has been extended to us by those already engaged in this department of medical effort. Our brethren of the *corps editoriale* throughout the Union, with but one or two exceptions, have honored us by exchanging; an act of courtesy, which, for obvious reasons, delicacy required, should be entirely unsolicited on our part. But in addition to this, nearly all have taken occasion to express their friendly feelings and wishes in a way which claims our especial acknowledgements. We beg to assure them that we appreciate their kindness, and fervently trust we shall not disappoint the favorable expectations which they are pleased to express.—There is one point of view, however, in which the favors of our contemporaries occasion us not a little dissatisfaction. We find constantly in our exchange journals a variety of matter, of which we desire to avail ourselves for the gratification and advantage of our readers, but which our contracted limits compel us to forego. In view of this difficulty, we look forward with impatience to the commencement of a second volume, when we hope to feel authorized to enlarge our journal sufficiently to do better justice to the sources of valuable and interesting information to which we have access. In the mean time, we propose to introduce to our readers, the Journals, severally, which we receive, with some remarks thereupon; and as it is far from our plan to wish to supersede others, even within the range of our immediate neighborhood, we trust that in so doing we may contribute somewhat to extend their circulation. Members of the medical profession are not, we fear, sufficiently alive to the importance of cultivating and encouraging medical, periodical literature. In neglecting this department, we sincerely believe they are not only impolitic as regards their individual interests, but the effect is to limit the usefulness of one of the most efficient means of elevating the char acter and improving the condition of the profession—but of this topic we shall speak at some future time. In pursuance of the plan just mentioned, we proceed to notice in this number the *American Journal of the Medical Sciences.*

This periodical is published quarterly, each number embracing at least 264 large and closely printed pages. If we mistake not, this is the oldest Medical Journal now extant in our conntry. It has been in existence 26 years. It was edited formerly by Prof. Chapman, so long the distinguished teacher of medicine in the University of Pennsylvania. For many years past it has been under the editorial charge of Isaac Hays, M. D., in whose hands it may be said, without invidiousness, to have become the most prominent representative of American, medical, periodical literature, both at home and abroad. It embraces original communications from members of the profession in every portion of the country; analytical reviews, and bibliographical notices; and a compendium of medical intelligence gathered from the periodicals of different countries. In addition to the well known industry and ability of its accomplished editor, it receives assistance from a large number of collaborators, who are among the most eminent in the profession. The long established character of this work renders it supererogatory to enter upon any eulogiums addressed to medical men.

Aside from the quarterly publication, a monthly is connected with it, of 32 pages, devoted to general medical intelligence, and the republication of standard works. The latter is sent gratuitously to those who subscribe for the larger periodical, the terms of which are five dollars per annum. It is published by the well known medical publishers, Lea & Blanchard, Philadelphia, Pa. [TO BE CONTINUED.]

